# Magnetically Driven Bionic Millirobots with a Low-Delay Automated Actuation System for Bioparticles Manipulation

**DOI:** 10.3390/mi11020231

**Published:** 2020-02-24

**Authors:** Xue Bai, Dixiao Chen, Wei Zhang, Heulin Ossian, Yuanyuan Chen, Yanmin Feng, Lin Feng, Fumihito Arai

**Affiliations:** 1 School of Mechanical Engineering & Automation, Beihang University, Beijing 100191, China; baixue041104@163.com (X.B.); chendixiao@buaa.edu.cn (D.C.); benxiaoheng534@buaa.edu.cn (W.Z.); Heulin.ossian@gmail.com (H.O.); chenyuanyuan0526@sina.com (Y.C.); feng_yan_min202@126.com (Y.F.); 2 Beijing Advanced Innovation Center for Biomedical Engineering, Beihang University, Beijing 100083, China; 3 Department of Micro-Nano Mechanical Science & Engineering, Nagoya University, Nagoya 464-0814, Japan; arai@mech.nagoya-u.ac.jp

**Keywords:** bionic robots, cell manipulation, magnetic control

## Abstract

This paper presents a semi-automatic actuation system which can achieve bio-particles tracking, transportation, and high-precision motion control of robots in a microfluidic chip. This system is mainly applied in magnetically driven robots. An innovative manta ray-like robot was designed to increase stability of robots in a non-contaminated manipulation environment. A multilayer piezo actuator was applied to generate high-frequency vibration to decrease the friction between robots and the glass substrate. We also set up a user-friendly GUI (Graphical User Interface) and realized robot tracking and predetermined trajectory motion through excellent algorithms using Python and C++. In biotechnology, precise transportation of cells is used for the enucleation, microinjection, and investigation of the characteristics of a single cell. Being optimized, the parameters of the robot can effectively reach 10 µm in actuation precision and a maximum actuation speed of 200 mm/s.

## 1. Introduction

In cellular study, especially the manipulation of bioparticles (e.g., cells and biological tissue), significant process has been made to achieve automatic high-throughput operations [1,2]. Manipulation of individual living cells represents a practicable technology that plays an important role in various biological applications. In particular, cell microsurgery proposes a feasible solution for cell cloning [3,4], early detection of cancer [5], etc. Numerous microdevices and micro/nanorobots have been designed to accomplish precise cell manipulation or measure properties of microorganisms. Chunlin Zhao et al. used a programmatic control method of cell oocyte nuclear injection to increase the blastocyst rate effectively [6]; Xinyu Liu presented a vision-based cellular force sensing approach, including a microfabricated elastic cell holding device and a sub-pixel visual tracking algorithm which can decrease forces to 3.7 nN during microrobotic mouse embryo injection [7]. In addition, many driving methods such as chemical reaction [8], acoustic waves [9], optoelectronics [10] and the magnetic field [11], are chosen for different reasons to manipulate microrobots. Among them, the magnetic field is a safe and powerful tool for its biocompatible and broad output force range [12]. High-output force is necessary for implementing tasks including cutting and puncture, as denucleation cannot be done if the zonal pellucida is not pierced. Therefore, we focused on magnetically actuated robots for cell transportation and cell sorting.

The magnetic field generated by Helmholtz coils and helical magnetic microrobots have been widely used for many studies and biological applications [13]. Various feasible methods have been used to fabricate micro/nano-scale helical robots, such as 3D direct laser writing [14], biotemplated synthesis [15], self-scrolling [16] and 4D printing technology [17]. Helical microrobots actuated by rotating magnetic fields could be used for drug loading in low Reynolds number blood environment [15,18], owing to their capability of transforming the rotation around the helical axis into a translational motion along the helical axis. Helmholtz coils or their improved versions can also be used to actuate microstructures with different magnetized directions in every part [19,20]. These deformable robots present an impressive idea for future application in minimally invasive medicine, directed particle transport and self-assembled swarm robots. However, the magnetic field generated by coils loses its advantage compared with permanent magnets in cell manipulation and particles transportation in vitro for its low magnetic field intensity to generate enough output force and high energy consumption [21]. Therefore, many biological applications such as loading, sorting, and droplet generation can be done properly with permanent magnets.

In 2011, Masaya Hagiwara et al. presented a novel driving method for a microrobot actuated by permanent magnets in a microfluidic chip. A piezoelectric ceramic was applied to induce ultrasonic vibration, which significantly reduced the effective friction on a magnetically driven microtool (MMT) [22]. Much work has been done with the help of the MMT. Feng et al. achieved the on-chip enucleation of bovine oocytes using a MMT with better operation speed, greater cutting precision, and potential for repeatable enucleation [23]. Feng et al. achieved positioning accuracy of less than 1 μm and the response speed and the output force were also significantly improved [24]. Tomohiro Kawahara et al. proposed a MMT with 5 mm-wide force sensors in a microfluidic chip by permanent magnets so that they can locally stimulate the microorganisms with the desired force within the stable environment of the closed microchip [25]. Qiang Zhou et al. achieved donor cell suction, delivery, and injection in a mammalian oocyte on a microfluidic chip with a milli-scale MMT [26]. In summary, the MMT is an ideal and prospective tool that can assists researchers to do lots of cell manipulation.

More work could be done easily if the dimension of the MMT would be smaller. The microfluidic chip provides an ideal confined space to biotechnology research for its low contamination risk and high repeatability. In addition, the microfluidic chip can establish a well-controlled micro-environment for manipulating fluids and particles. However, the centimeter-scale robots are hard to be integrated into a microfluidic chip because of their large dimensions compared to cells [27]. Milli/Micro-scale robots are essential because them more delicate cell research such as organelle extraction and gene injection can be done. 

In this study, a semi-automated actuation system for magnetically driven millimeter-scale robots with 3 DOF (degree of freedom) (X-axis movement, Y-axis movement, Z-axis rotation) was designed. The system for cell transportation introduced a piezoelectric ceramic to reduce the friction between robots and the glass substrate significantly. In addition, all operations were conducted in a microfluidic chip and the robot was magnetically actuated, providing a solution that achieved repeatability, precision and high-speed manipulations. A program incorporating three key features: GUI, image feedback and actuation was designed using Python and C++ on Microsoft Visual Studio. This program is user-friendly, and accessible by any operator and allows an enhanced visual experience of the experiments while allowing complex and accurate moves, either by semi-automatic motion (using the tracking features in our previous work [24]), or by direct inputs. In the system, we chose the canny filter, which showed the greatest stability and accuracy for the various possibilities in terms of object detection, classification and tracking. We manufactured a manta ray-like robot actuated by three permanent N38 magnets. This bionic robot showed great capabilities in terms of speed, accuracy, repeatability and low friction of the movement.

**Figure 1:**
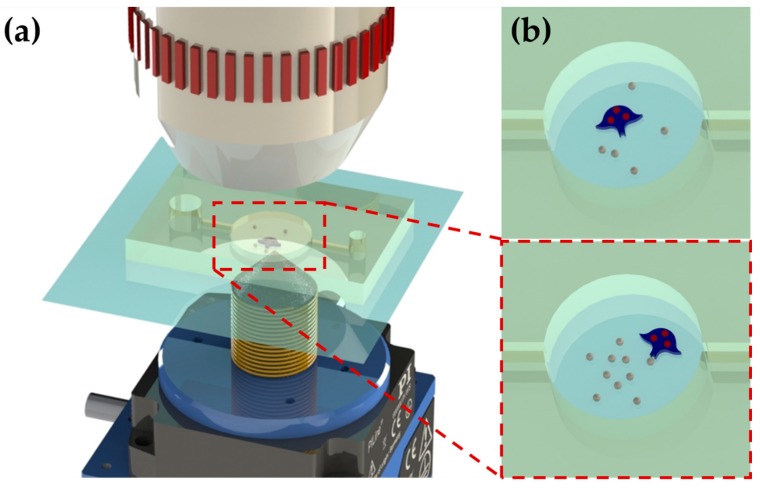
(**a**) Robot actuation system. (**b**) The process of moving particles into a set shape.

Figure 1 shows a conceptual overview of the proposed robot actuation system. The robot can be actuated by external permanent magnets to move or rotate. The robot can be designed to complete complicated tasks such as moving particles into a set shape.

## 2. Materials and Methods 

### 2.1. System Design

The principle of magnetic field generation of the MMT actuation system is different to that of electro-magnetic coils. The gradient of the magnetic field and the magnetic field intensity produced by permanent magnets are larger compared to those of electromagnetic coils. Any magnetic substance in a magnetic field will be subject to the magnetic field force shown in Equation (1) [28].

(1) $${\vec{F}}_{\mathrm{mag}}\;=\;\frac{{4\pi a}^{3}}{3}\frac{\mu_{0}\chi }{\left(1+\chi /3\right)}\vec{H}\frac{d\vec{H}}{d\vec{x}}\;=\;\frac{{2\pi a}^{3}}{3}\frac{\mu_{0}\chi }{\left(1+\chi /3\right)}\nabla \left({\left|\vec{H}\right|}^{2}\right)$$

where a is the radius of a nanoparticle [in meters], $\mu_{0}{\;=\;4\pi \;\times \;10}^{-7}N/A^{2}$ is the permeability of vacuum, χ is the magnetic susceptibility, $\vec{H}$ is the magnetic intensity [Amperes/meter], and ∇ is the gradient operator [with units 1/m]. According to the equation, we conclude 3 points. First, a varying magnetic field is required to create a magnetic force. Second, the magnetic force depends on the intensity and the gradient of the magnetic field. Third, the direction of the magnetic force is from a low magnetic field intensity area to a high magnetic field intensity one. Therefore, the MMT will experience a force that depends upon the magnetic field and the field gradient applied at its location.

**Figure 2:**
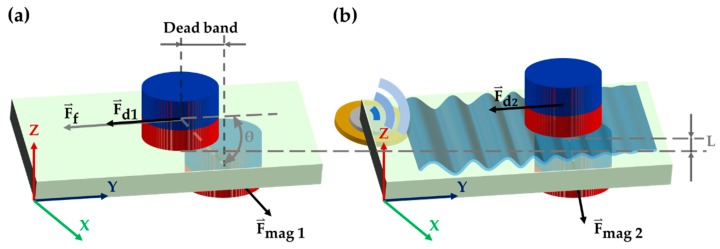
(**a**) Dead band caused by friction between magnetically driven microtool (MMT) and substrate. (**b**) Friction reduction principle of piezoelectric ceramics.

Magnetic robots in the magnetic field will follow the movement of external permanent magnets to achieve functions such as translation and rotation. As shown in Figure 2a, except for magnetic force, the MMT is also subject to liquid resistance (${\vec{F}}_{d1}$) and friction between the magnet and the glass substrate (${\vec{F}}_{f}$). ${\vec{F}}_{f}$ is what hinders the movement of robots. Moreover, the dead band which is caused by ${\vec{F}}_{f}$ deteriorates the positioning accuracy of robots and the controllability of the robot on a chip. In order to get greater output force (${\vec{F}}_{\mathrm{op}}$) and higher positioning accuracy, it is necessary to reduce the liquid resistance (${\vec{F}}_{d1}$) and the friction (${\vec{F}}_{f}$). The output force of the robot is shown in Equation (2).

(2) $${\vec{F}}_{\mathrm{op}}{\;=\;\vec{F}}_{\mathrm{mag}}\;\cdot {cos\theta \;-\;\vec{F}}_{f}\;{-\;\vec{F}}_{d}$$

Ultrasonic levitation has been used for levitating small particles by creating a standing wave field. As shown in Figure 2b, a thin water film caused by ultrasound lifts the robot up from the glass substrate, bringing lubrication and reducing static friction. Therefore, the levitation height L can be expressed as [24]:

(3) $$L=\;\frac{1}{2k}\sqrt{\frac{\rho_{0}}{2\sigma g}U_{1}}$$

where *k* is the wave number, i.e., *k* = ${\omega /c}_{0}$ (where ω is the angular frequency of piezo ceramic vibrations in the vertical axis and $c_{0}$ is the sound speed of infinitesimal amplitude), $\rho_{0}$ is the medium density, σ is the surface density, i.e., σ = m/$S_{0}$ (m is the mass and $S_{0}$ is the bottom area of the levitated object) and $U_{1}$ is velocity amplitude. In the experiment, a buzz piezoelectric ceramics was pasted to the glass substrate to generate ultrasonic waves, which levitated the robot with a planar bottom surface.

### 2.2. Robot Design

**Figure 3:**
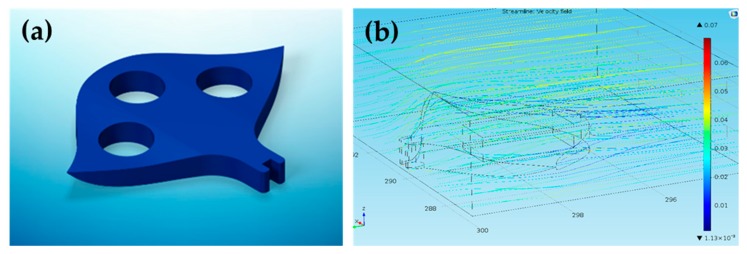
(**a**) Concept drawing of the manta ray-like millirobot. (**b**) Simulation result of robot. (unit: mm/s).

Our robot was designed to transport particles whose dimension varied from 50 um to 500 um. As shown in Figure 3a, the robot was designed with a tiny operator on its tip. For a cylindrical permanent magnet in a magnetic field generated by a cylindrical magnet, it will be subject to a magnetic force which can be approximated by the formula:

(4) $$F\left(x\right)\;=\;\frac{{\pi \mu }_{0}}{4}M^{2}R^{4}\left[\frac{1}{x^{2}}+\frac{1}{{\left(x\;+\;2t\right)}^{2}}-\frac{2}{{\left(x\;+\;t\right)}^{2}}\right]$$

where R is the radius of the cylindrical magnets, t is the height of the magnets, M is the magnetization, and x is the gap between them. According to Equation (4), three magnets in the robot are subject to a magnetic force in the magnetic field. A manta ray-like millirobot was designed and fabricated through 3D printing, with an overall size of 6 mm in width, 4 mm in length and 0.5 mm in thickness. Figure 3b shows the hydrodynamic simulation result of the millirobot. Benefiting from the small dimension, the robot brings little interference to environment and cells when it moves.

**Figure 4:**
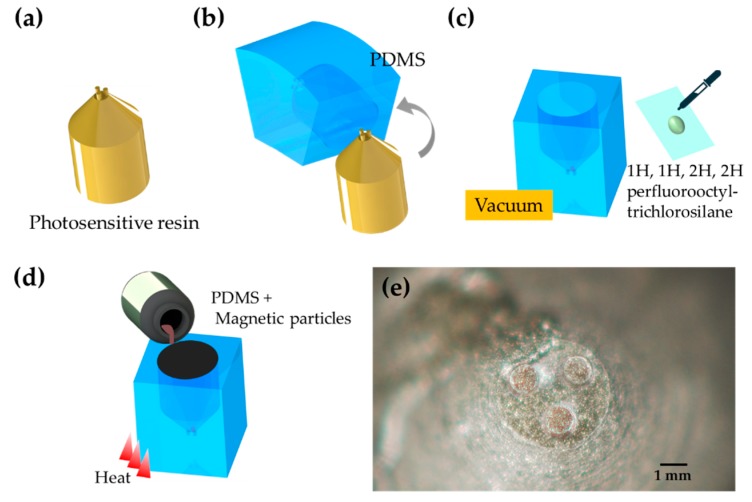
(**a**–**d**) The fabrication process of the external magnets. (**e**) The photograph of the fabricated magnet.

At the same time, in order to control the robot more precisely, we required the external permanent magnets to generate a magnetic field, which has a larger intensity in the area of 3 magnets. An external magnet with 3 cylindrical tips was used to focus the magnetic field on the area where the magnets of the robot were. The fabrication process of the external magnets is shown in Figure 4a–d and the photograph of the fabricated magnet is shown in Figure 4e, respectively. The external permanent magnets were attached to a manipulator which contained two linear micro-motion stages and a rotation stage. 

### 2.3. GUI Design

We wrote a program using Python and C++ to design a GUI to control the movements of these stages simultaneously in real time. A live video of the operating area was also contained in the interface to assist us recognizing and locating the target objects rapidly. In our previous work, we have succeed in positioning a single oocyte at predetermined locations [24]. Based on the architecture of the GUI shown in Table 1, the development of the proposed system is split into two entities. The first one, developed using Python on Anaconda-Spyder (Anaconda Inc., Austin, TX, USA) virtual environment, using the OpenCV library (Willow Garage Inc., Palo Alto, CA, USA) as well as RapidJson (Shenzhen city Tencent computer system Co., Ltd., Shenzhen, China) and Websocket (The Internet Engineering Task Force, Wilmington, DE, USA) plugins, will define both the GUI and its back end, dealing with the user interaction, the visual tracking and the various parameters adjustment necessary for the program to fit the hardware specifications. The second part of the program will be developed using C++ on Microsoft Visual Studio, based on the proprietary library provided by PI for its platform, using Websocket and RapidJson plugins as well for communications purposes between the two parts.

**Figure 5:**
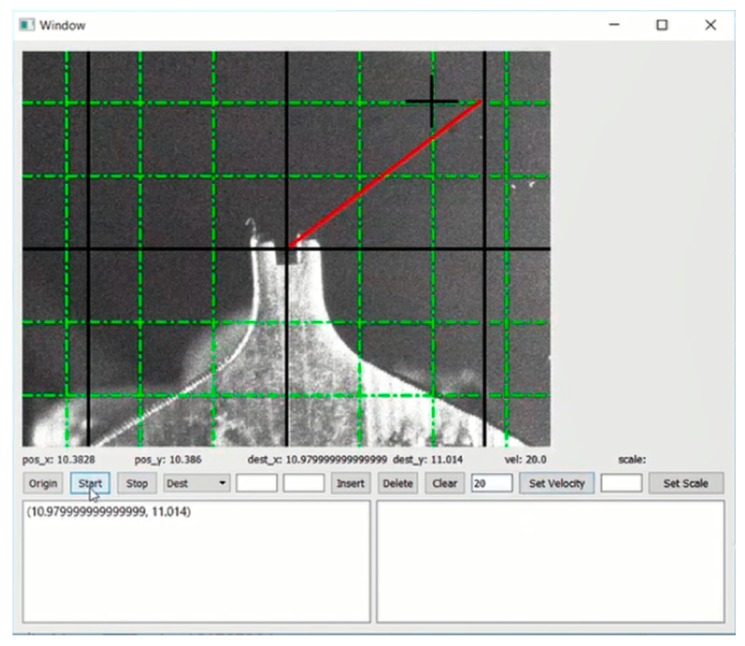
Real-time feedback.

In the GUI’s back end classes, the thread initializes the video and its various parameters such as the encoding of the frames, the frame rate and the resizing of the frames. The frame rate is fixed at 20 frames per second to ensure the programs run without hurdles. The frame is resized at a 480 × 640 pixel format, out of a consideration for the implementation in the GUI and the original format coming from the FlyCap camera (FLIR^®^ Systems Inc., Wilsonville, OR, USA). The thread will also handle the basic video tracking features. The processing of the frame through Gray Scaling, Blur, Canny detection and Hough Circles is thus achieved. Besides, the controller lists and runs all the functions called by the interaction of the user with the GUI. We adopted the setting that any action on the listed widget will trigger a function (attribute of the class) that’ll make a calculation. Specifically, the key feature of the controller in our program is the handler of the mouse press. This attribute is a hardcoded one that will deal with any action performed through the mouse or the pad. The idea is that every click on the video feed of the GUI triggers an action, but a click on another segment leaves it unscathed. The sever class can handle the detection and connection to it, followed by a bidirectional exchange of data. The data exchange is based on a Websocket (The Internet Engineering Task Force, Wilmington, DE, USA) with RapidJson (Shenzhen city Tencent computer system Co., Ltd., Shenzhen, China) packages protocol to make the connection fast and reliable for its local definition regarding the HTTP protocol. The server is defined in 4 parts including Listen, Send, Receiver and Disconnect. We set the frequency to 20 ms at which the platform would be sending data when the user interacted with the GUI. The GUI is shown in Figure 5.

## 3. Experiments and Results

### 3.1. System Setup

**Figure 6:**
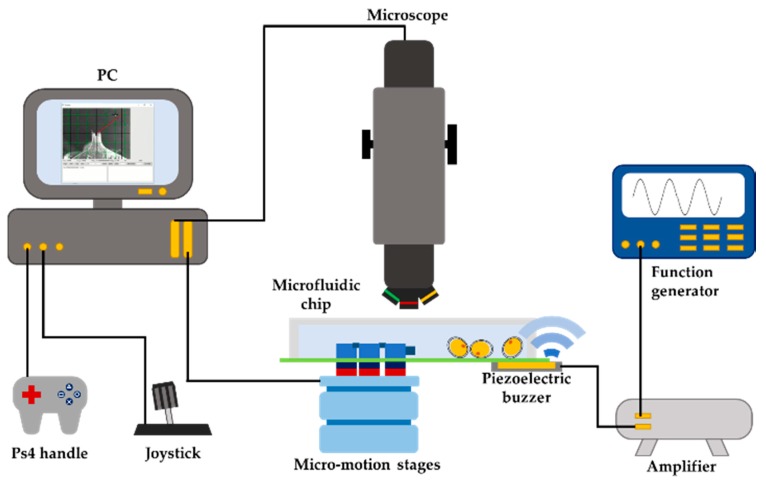
Overview of the magnetically driven microrobot system.

Figure 6 shows an overview of the robot system, consisting of the manipulation platform, and the observation and actuation systems. A 3D digital microscope (MXB-2500REZ, Hirox Co., Ltd., Tokyo, Japan) is used to observe surface topography and the manipulation better. A personal computer is connected to the microscope to get real-time and stereoscopic images. The microfluidic chip is fixed on a stage and 2 linear micro-motion stages (U-521.23 PILine® XY Stage, Physik Instrumente GmbH & Co., Karlsruhe, Germany) and 1 rotation micro-motion stage (U-624.03 PILine^®^ XY Stage, Physik Instrumente GmbH & Co., Karlsruhe, Germany) can be controlled by Ps4 or joystick or GUI separately. The function generator (Wave Station 2012, LECROY, New York, NY, USA) generates a sine wave to a high-voltage amplifier (ATA-2042, Agitek Co., Xi’an, China). This output signal is sent to actuate the piezoelectric ceramics (TAK050510, TAISEI Co., Ltd., Chichibu, Japan).

In the fabrication process of the robot, we ordered the mold which was made through 3D printing (S140, BMF Material Technology Inc., Shenzhen, China). Printing accuracy is up to 10 µm. The mixture of PDMS (polydimethylsiloxane) and 5 µm NdFeB particles (MQPF, Magnequench Co., Tianjin, China) was cured to fabricate the robot.

### 3.2. Beads Transporting Experiment

In the experiment, we used microbeads to replace the cell and accomplished the transportation function. The piezoelectric ceramics were applied to the square wave whose frequency and amplitude were 1.8 kHz and 30 Vpp, respectively.

**Figure 7:**
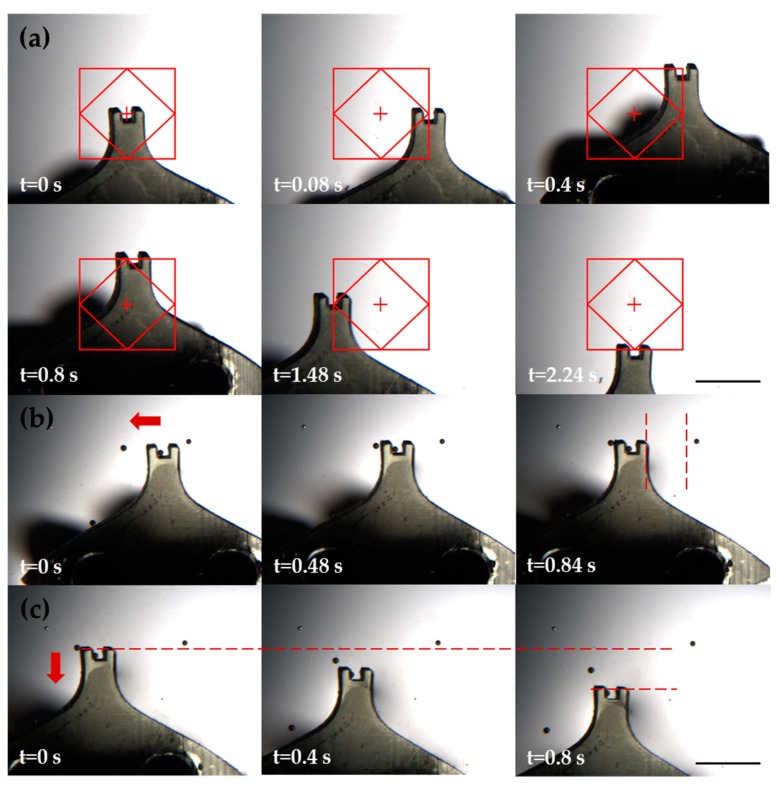
Beads transporting experiments of the robot. (**a**) Robot moves along a predetermined trajectory. (**b**) Robot moves in the X-axis direction to transport a particle. (**c**) Robot moves in the Y-axis direction to transport a particle (150 μm). Scale bar: 1 mm.

As shown in Figure 7a, the robot was set to move in a predetermined trajectory automatically. This movement was achieved by running the software to control the stage. The sequences of the whole movement process are contained in supplementary information (Supplementary Material Video S1). The experimental path was then retrieved through a Point Grey Fly Cap camera at 24 fps and compared to the input path in the XY grid of the GUI. Figure 7b shows the process of the robot transporting a particle in the X-axis direction. The movement of the robot can also be controlled by joystick and a Ps4 handle.

**Figure 8:**
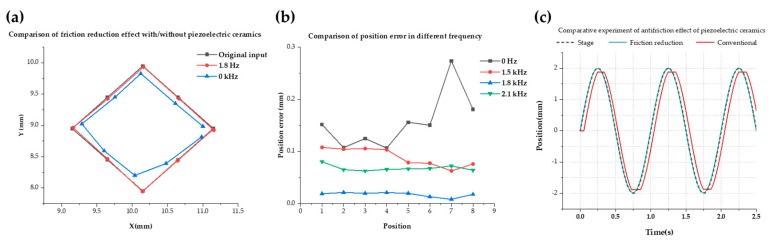
(**a**) Comparison of friction reduction effect with/without piezoelectric ceramics. (**b**) Comparison of position error at different frequencies. (**c**) Comparative experiment of antifriction effect of piezoelectric ceramics.

The effect of the friction reduction of the piezoelectric ceramics was confirmed by the second experiment results that are shown in Figure 8a–c. As shown in Figure 8a, the experiment involved actuating the robot over a square patter (8 steps) with its 4 corners being in (9, 9), (10, 8), (11, 9), (10, 10). The piezoelectric ceramics were connected to a wave generator (10 Vpp, sinusoidal signal, frequency within [1 kHz; 15 kHz]; offset = 0 V, phase 0°) and a voltage amplifier (voltage within [0; 4]) which allowed us to provide a voltage ranging from 0 V to 80 V to the ceramics. The piezo was vibrating in a vertical mode at an amplitude ranging from 1 micron to 3 microns at its resonance frequency (13 kHz +/− 0.2). Figure 8b shows that under different frequencies, the reduction of position error is discrepant. When the piezoelectric ceramics were under a frequency, which was the resonance frequency of the microfluidic chip, the levitation height of the robot was the greatest. As shown in Figure 8c, the following response ability of the robot was evaluated by us through a simple 1 DOF movement. The stage moved in a sine wave of 1 Hz and the stroke for 2 mm. In the conventional driving method (without vibration), movement of the robot can hardly achieve precise conduction. However, applied vibration decreased the time for response and positioning accuracy (mean error = 9.3 μm).

## 4. Conclusions

The manipulation of cells requires perfect accuracy and repeatability as well as a short time cycle for large-scale application. Compared to previous design of MMT, we made a relatively small robot, which could be compacted into a microfluidic chip. In the meantime, a user-friendly GUI for automatic control was developed. The GUI is accessible to any operator and allows an enhanced visual experience for the experiments. Moreover, it has enabled the complex and accurate moves, either by semi-automatic motion, or by direct inputs. This robot, coupled with the previously designed actuation and tracking program, demonstrated great capabilities in terms of speed, accuracy and repeatability of the movement. The effect of the friction reduction of the piezoelectric ceramics also has been verified, which can bring beneficial to the high accuracy in cell manipulation in the future. The system can perform precise motion that is independent from speed, reaching 200 mm/s with the same accuracy of 10 μm. 

## Figures and Tables

**Table 1:** Overall architecture of the GUI (Graphical User Interface).

Live Video Feedback	Data Display	Data Input
Grid 1 mm step	Current position	Coordinates
Origin platform	Next position	Velocity
Current position	Velocity	Scale
Path followed	Scale	Start
Path incoming	Tracked objects coordinates	Stop
Tracked objects	List of awaiting position	Actuation mode
		Clear all inputs
		Change Origin

## Supplementary Materials

The following are available online at https://www.mdpi.com/2072-666X/11/2/231/s1, Video S1: Movement experiments.
